# Proprietary Pharmacists and State Medical Association Journals

**Published:** 1905-05

**Authors:** 


### PROPRIETARY PHARMACISTS AND STATE MEDICAL ASSOCIATION JOURNALS.

The State Medical Association's journal, to begin July 1, will have to dance to the music of the Chicago Octopus (the *Journal A. M. A.*) and its ethics, as to *proprietary medicines* to be advertised will be prescribed by that organ, or, rather, by its ex-homeopathic editor. He is behind the State Association journal movement in all the States, and in only one State (California) is such a journal paying expenses, and the California journal is bucking against said octopus, and its editor. Simmons (ye editor) "draws the line" at all proprietary medicines the formula of which is not published, or mention is made of any disease in which it is recommended, and under that autocratic ruling,—which all State journals must obey or be "unethical,"—Antiphlogistine, Antikamnia, Ammonol, Unguentine, Ergo-Apiol (Smith), Pepto Mangan (Gude), Tongaline, Listerine, Peter's Peptic Essence, Arsenauro, Glycerophosphates Compound, Ecthol, Glycothymoline, Upjohn's preparations, and many other of the best preparations owned by the most liberal and prompt-paying firms, will have to be excluded. Getting $2400 worth of cash advertising to start with, after obeying the ethics of ye Octopus, is easier said than done. Does ye Octopus think these parties are going to give their trade-marked and protected business to every substitutor in Texas? Not much.

THE NEW EXAMINING BOARDS. On May 10th the Governor, from lists of eighteen from each State Association, appointed the three licensing boards as follows:

State Board of Medical Examiners: Dr. J. T. Wilson, Sherman; Dr. S. R. Burroughs, Buffalo; Dr. T. J. Bell, Tyler; Dr. S. T. Turner, El Paso; Dr. D. J. Jenkins, Daingerfield; Dr. M. M. Smith, Austin (members of the old board, reappointed); Dr. T. T. Jackson, San Antonio; Dr. R. T. Morris, Houston; Dr. A. C. Scott, Temple.

Eclectic Medical Examiners: G. W. Johnson, of San Antonio; C. D. Hudson, of Waco; M. E. Daniel, of Honey Grove; J. N. White, of Queen City; E. L. Fox, of Houston; G. Hebling, of Bonham; Charles Dowell, of Ennis; L. S. Downs, of Galveston, and T. F. Chandler, of Gainesville.

Homeopathic Medical Examiners: W. R. Owen, of San Antonio; E. B. Stiles, of Waco; T. J. Crowe, of Dallas; J. R. Pollock, of Fort Worth; F. L. Griffith, of Austin; A. W. Thatcher, of Denison; C. E. Johnson, of Sherman, and S. W. Cohen, of Waco.

---

A COPY of this Souvenir Edition can be had for 20 cents, silver or stamps. Limited edition. Exchanges will oblige me by a mention.

